# 8.Q. Workshop: WHO’s activities to strengthen the evidence of public health and social measures

**DOI:** 10.1093/eurpub/ckac129.532

**Published:** 2022-10-25

**Authors:** Organised by: WHO, Chair persons: Ramona Ludolph (Switzerland), Ryoko Takahashi (Switzerland)

## Abstract

Public health and social measures (PHSM) are key to reducing the spread of infectious diseases like COVID-19, especially in the early stages of an outbreak. PHSM include non-pharmaceutical actions that individuals, communities, and governments take to reduce person-to-person contact and/or make them safer. PHSM reduce the pressure on the health care system to allow for the continuation of essential services and buy time for the development and dissemination of treatments and vaccines. While a combination of PHSM has proven to be effective in reducing transmission during the COVID-19 pandemic, the evidence on the relative effectiveness and broader health, social and economic impact of individual interventions is still scarce. However, PHSM packages such as lockdowns can have severe unintended consequences for individuals and societies including economic hardship, decreased mental health and wellbeing and exacerbated social and health inequity; therefore, precision in PHSM decisions and implementation is needed. This requires a strengthened evidence base as well as tools that support countries in making balanced decisions about PHSM with the best possible cost-benefit ratio. WHO works with multisectoral partners to achieve effective and context- specific PHSM implementation while maximizing the benefits of PHSM and keeping their health, social and economic burden to a minimum and justifiable. In the WHO Regional Office for Europe, PHSM are a key pillar in the COVID-19 response. The office provides, amongst others, a PHSM monitoring dashboard to track the severity of implementation across all 53 Member States as well as a calibration tool and related capacity building to guide countries in their implementation decisions as the pandemic situation evolves. The WHO has further launched a global initiative in 2021 to measure the effectiveness and impact of PHSM and improve precision in future PHSM decisions and policies. The initiative aims at providing robust data and research evidence on PHSM through a global conceptual model and research agenda, a central research monitoring system and harmonized data collection mechanisms during health emergencies. It further seeks to integrate PHSM into emergency preparedness and response assessments and yield a decision-making tool to facilitate evidence-informed and context-specific PHSM implementation. This workshop will provide an overview of WHO's activities in advancing PHSM research and implementation for better decision-making during future emergencies. Three presentations by the WHO Secretariat at the regional and global levels will focus on the strategic approach and the main deliverables of the respective initiatives so far. The presentation by the University of Munich will provide in-depth insights into the conceptual model on PHSM. The workshop will actively solicit feedback from participants on planned activities and results gathered to-date.

**Key messages:**

• The lack of evidence on PHSM effectiveness and impact hampered an evidence-driven implementation approach during the COVID-19 pandemic.

• WHO works to achieve effective and context- specific PHSM implementation to maximize the benefits of PHSM while keeping their health, social and economic burden to a minimum and justifiable.

